# Supplementary material for: A tumorigenesis threshold for endogenous Myc revealed by dosage-compensation for Myc-haploinsufficiency in the absence of p53
Source: bioRxiv. 2025 Jul 31:2025.07.28.667174. Preprint. [Version 1] doi: 10.1101/2025.07.28.667174 (PMC12443044; doi:10.1101/2025.07.28.667174)

Supplementary Table 1.

Frequency of tumor types in *Myc*-Wt and *Myc*$^{+/-}$ *p53KO* mice

	Myc$^{+/-}$;*p53KO*		*Myc*-WT;*p53KO*	
	tumor frequency	median age onset (days)	tumor frequency	median age onset (days)
*All	91%	131	98%	91
Hemangiosarcoma	31%	105	41%	86
**Lymphoma	17%	153	23%	99
Hemangiosarcoma-+lymphoma	17%	111	20%	81
***Other	26%	83	15%	91

*4-7% death w/o tumor, **~80% thymic primary, ***adenocarcinoma,sarcoma embryonal carcinoma, PNET

Supplemental Figure 1.

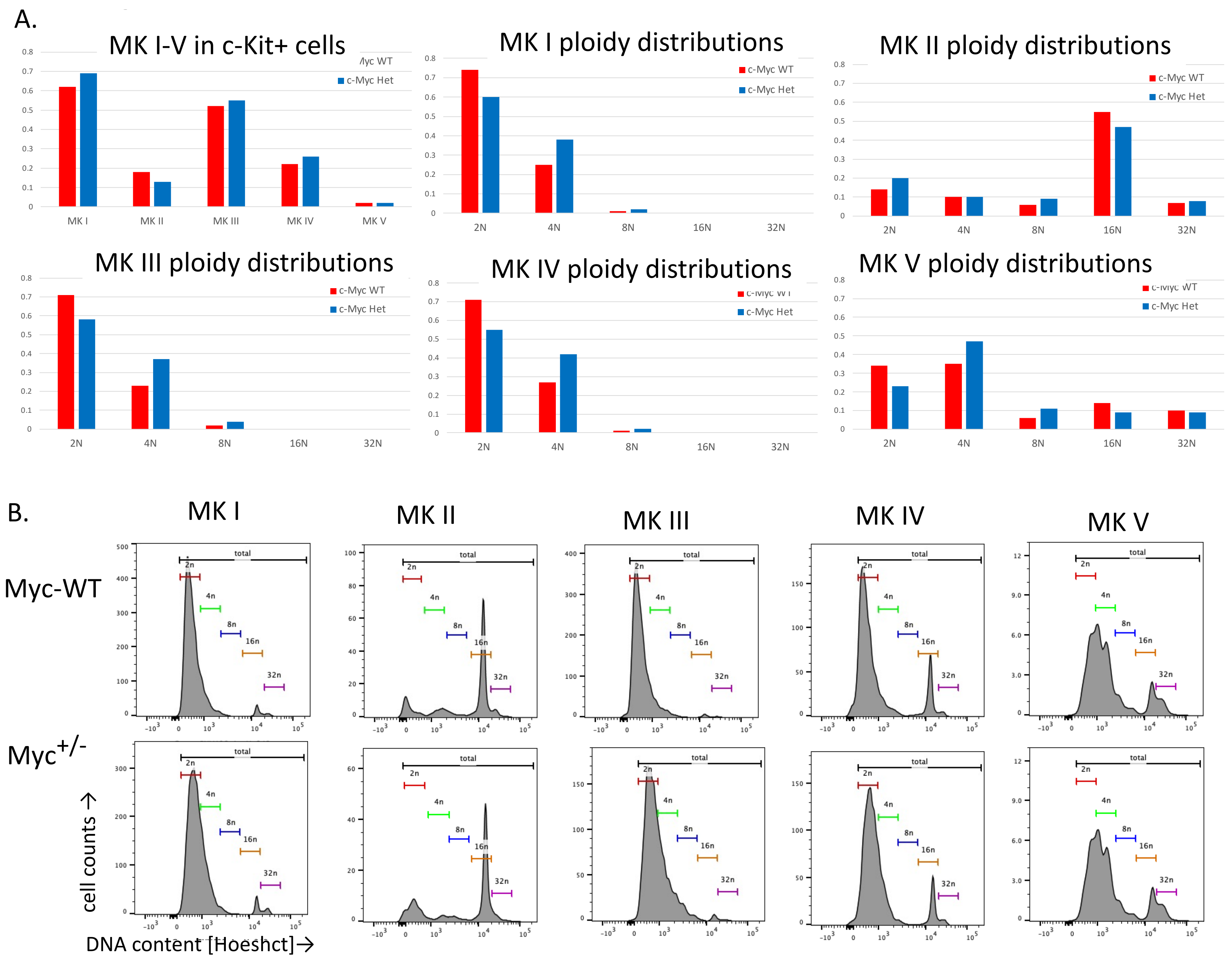

Supplemental Figure 2.

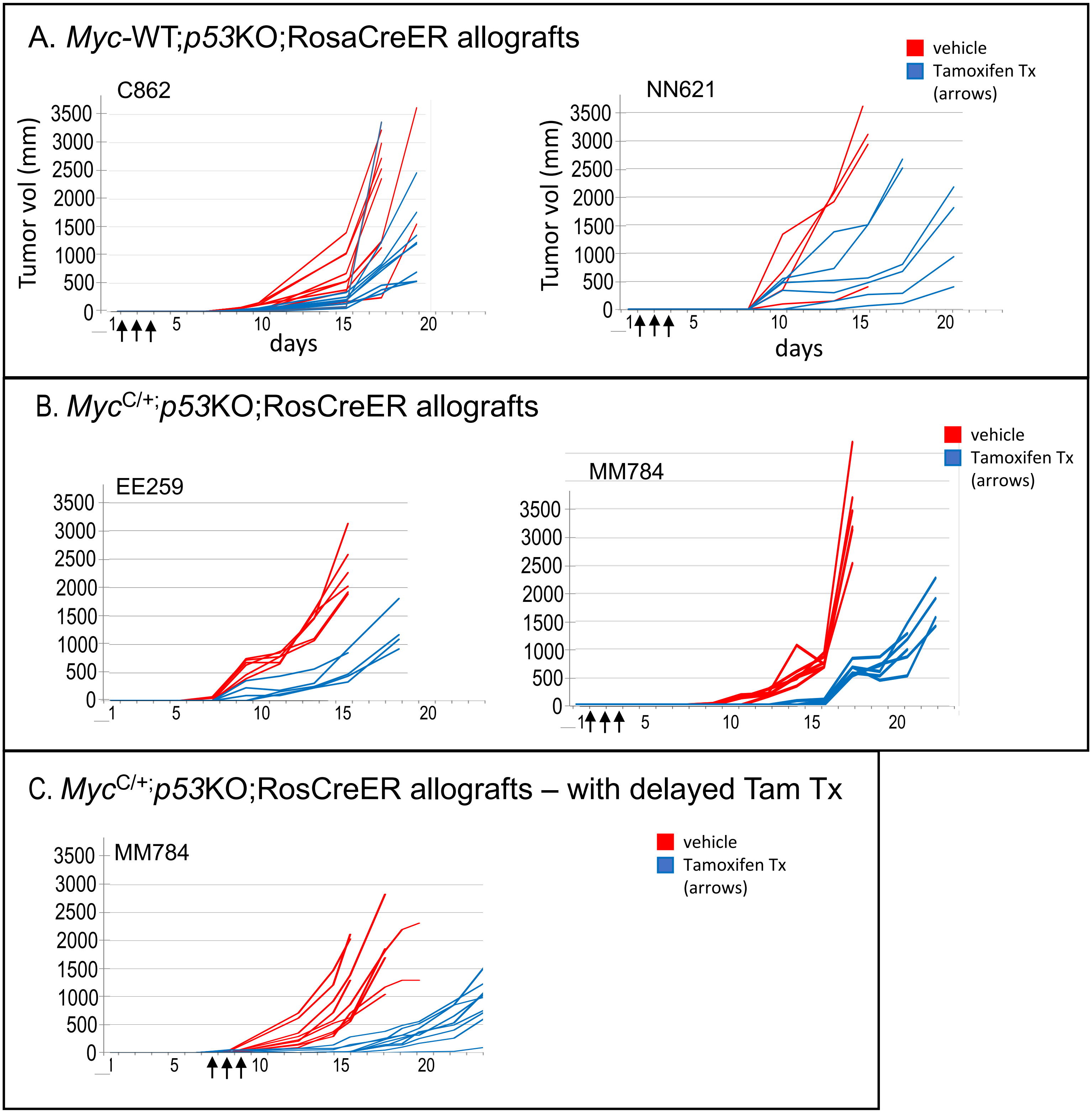

Supplemental Figure 3.

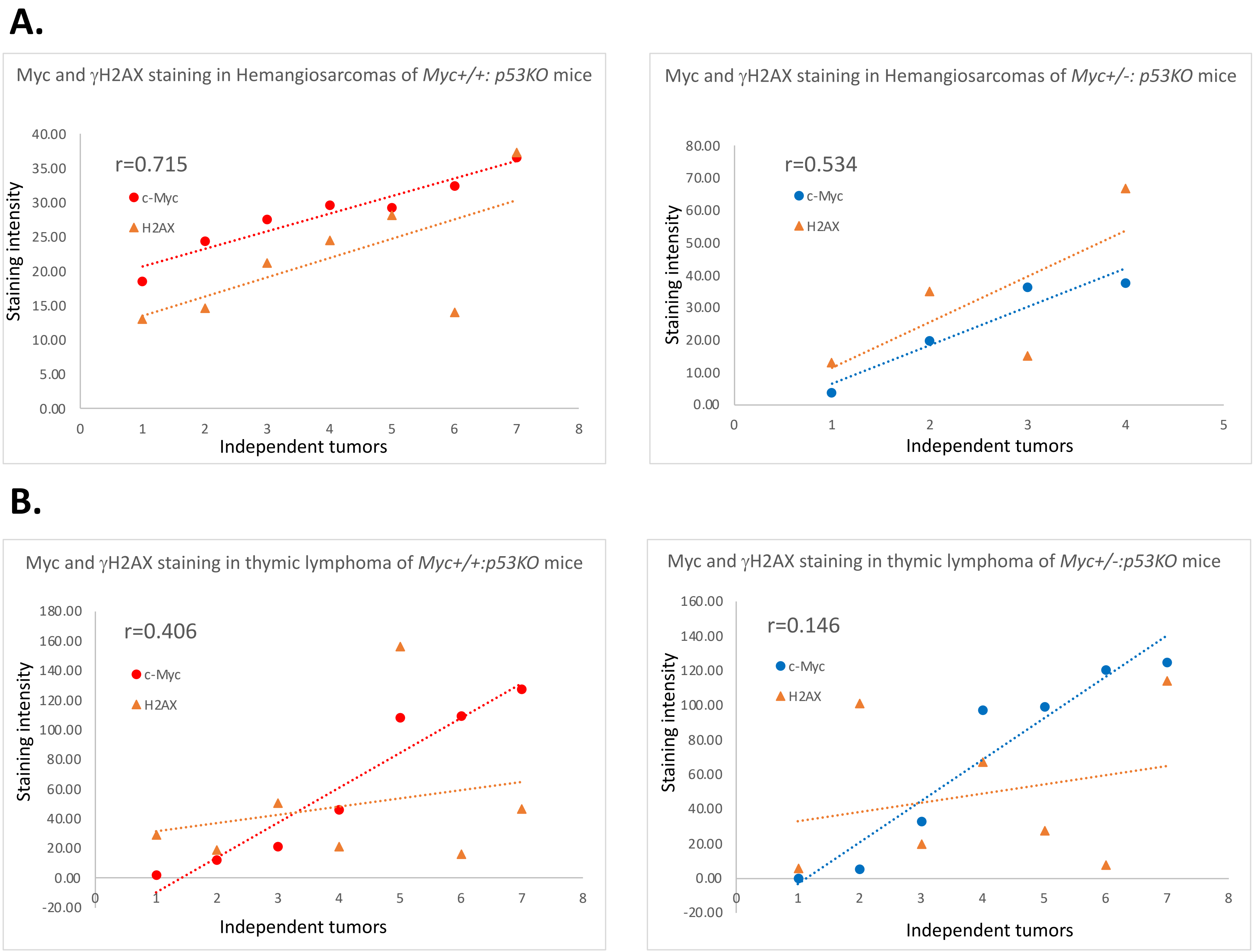

Supplemental Figure 4.

A.

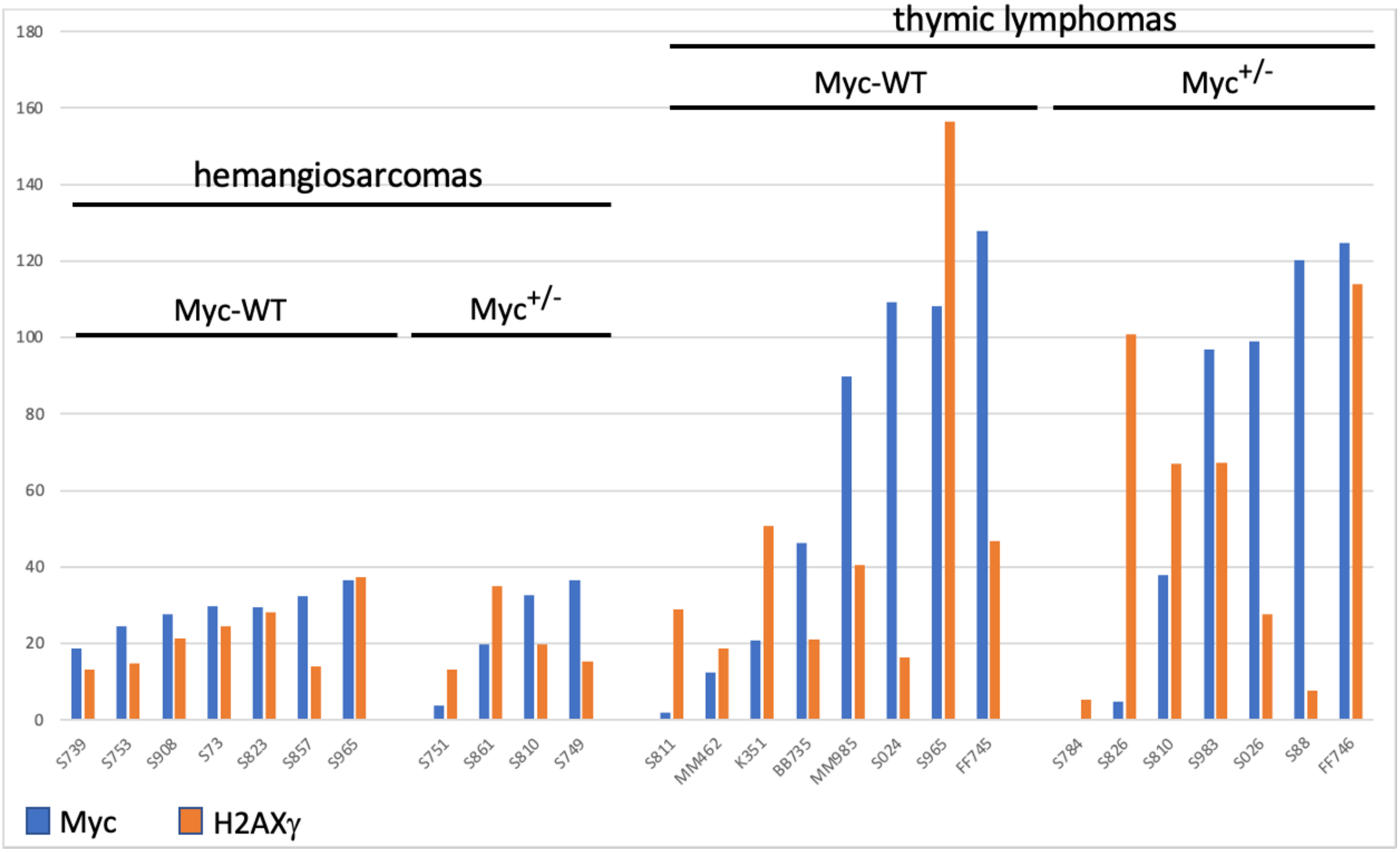

B.

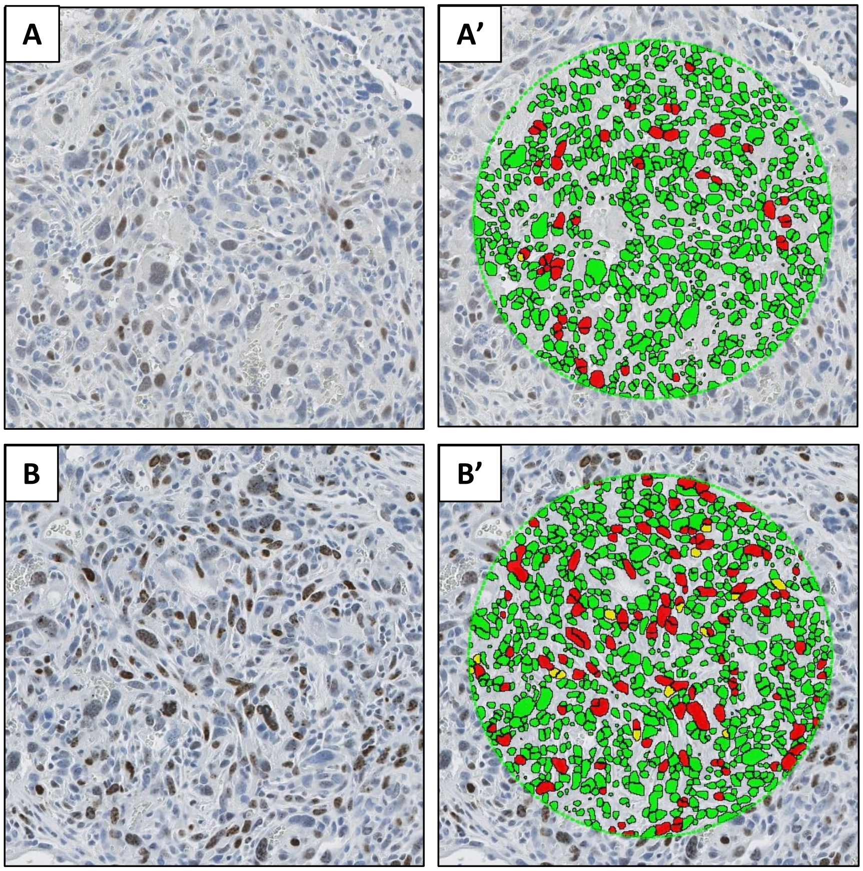

Supplemental Figure 5.

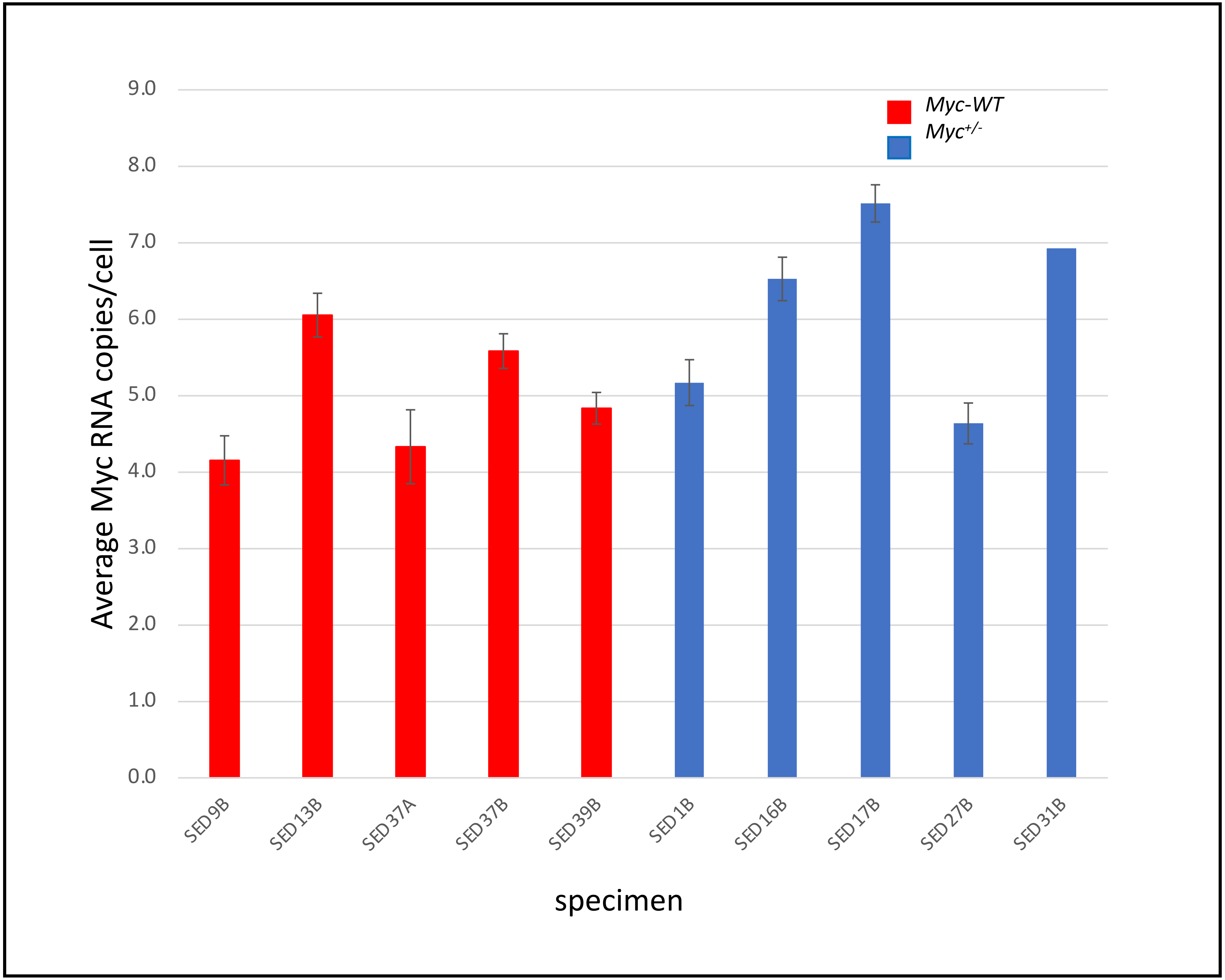

Supplement: 1 — Figure S1. Myc dosage reduction (Myc+/−) does not affect ploidy distribution in a non-neoplastic, normally polyploid population of maturing megakaryocytes. A. Bar graphs comparing distribution of megakaryocyte (MK) subpopulations (MKI-MKV) and the relative DNA content distributions (2N-32N) within a subpopulation for Myc-WT compared to Myc+/− bone marrow cells subjected to flow cytometry (from p53+/− mice; n=4 independent bone marrows from Myc-WT and from Myc+/− mice). No statistically significant differences in either subpopulation distributions or in DNA content profiles within a subpopulation were detected between Myc-WT and Myc+/− megakaryocytes by the 2-tailed t test at the 95% confidence level. B. Representative examples of DNA content profiles from flow cytometry of different megakaryocyte maturation sub-populations MKI-MKV from Myc-WT and Myc+/− mice. Figure S2. Acute reduction of Myc gene dosage in allografted, established p53KO thymic lymphomas impairs tumor growth. Primary data for graphs of mean tumor sizes over time, shown in Figure 6. Dissociated cells from Myc+/+ (C862 and NN621 tumors) or conditional MycC/+ (EE259 and MM784 tumors) p53KO;RosaCreER+ thymic lymphomas were injected subcutaneously in the flank of nude mice (ranging from 1–5×10^5^ cells) and treated in parallel with either tamoxifen (blue) or vehicle (red) for 3 consecutive days (arrows) beginning at either 1 day (A, B) or 7 days (C) after allografting. Each graph line represents growth plotted for an independent tumor inoculum into flank. A. Tumor allograft growth (mm3) for Myc-WT C862 and NN621 primary tumors, treated with vehicle or tamoxifen beginning 1 day after allograft. B. Tumor allograft growth (mm3) for MycC/+ EE259 and MM784 primary tumors, treated with vehicle or tamoxifen beginning 1 day after allograft. C. Tumor allograft growth (mm3) for MycC/+ MM784 primary tumor, treated with vehicle or tamoxifen beginning after a delay of 7 days after allograft. Figure S3. Correlation of M [file NIHPP2025.07.28.667174V1-supplement-1.pdf]
